# Ethical issues in the design and conduct of stepped-wedge cluster randomized trials in low-resource settings

**DOI:** 10.1186/s13063-019-3842-1

**Published:** 2019-12-19

**Authors:** Kaustubh Joag, Guillermo Ambrosio, Edgar Kestler, Charles Weijer, Karla Hemming, Rieke Van der Graaf

**Affiliations:** 1 Centre for Mental Health Law and Policy, Indian Law Society, Pune, India; 2 Epidemiological Research Center in Sexual and Reproductive Health, Guatemala City, Guatemala; 3 Rotman Institute of Philosophy, Western University, London, ON Canada; 4 Institute of Applied Health Research, University of Birmingham, Birmingham, UK; 5 University Medical Center Utrecht, Utrecht University, Julius Center, Utrecht, The Netherlands

**Keywords:** Stepped-wedge cluster randomized trials, Equipoise, Low-resource settings, Research ethics, Trial design

## Abstract

**Background:**

Stepped-wedge cluster randomized trials (SW-CRTs) are increasingly popular in health-related research in both high- and low-resource settings. There may be specific ethical issues that researchers face when designing and conducting SW-CRTs in low-resource settings. Knowledge of these issues can help to improve the ethical conduct of SW-CRTs in a global health context.

**Methods:**

We performed an ethical analysis of two studies using SW-CRT designs in low-resource settings: the Que Vivan Las Madres study conducted from 2014 to 2017 in Guatemala and the Atmiyata study conducted from 2017 to 2018 in rural parts of India. For both case studies, we identified and evaluated the classification of the study as research or nonresearch and the ethical issues regarding the justification of the design, including the delayed rollout of an intervention that had a promising effect.

**Results:**

In our case studies, some minor ethical issues surfaced about the registration and stakeholder pressure on the order of randomization, but both included good justification for the design and delayed rollout. Our analysis did, however, demonstrate that careful consideration of the role of randomization and registration of the trials is important.

**Discussion:**

SW-CRTs can provide an opportunity for rigorous evaluation of interventions destined to be rolled out on the basis of limited evidence. Furthermore, in SW-CRTs, the underlying objective is often to provide a robust evaluation of the effectiveness for generalized dissemination, and this makes the SW-CRT no less a research study than any other form of cluster randomized trial.

**Conclusion:**

The design and conduct of stepped-wedge cluster randomized trials raises at least two ethical issues that need special consideration in both high- and low-resource settings: the justification for using the design, specifically the delayed rollout of the intervention to the control group, and the classification of the study as research or nonresearch. In our case studies, these issues did not seem to raise special ethical scrutiny in low-resource settings. Further ethical evaluation will hopefully result in specific ethical guidelines for the use of SW-CRTs in both high- and low-resource settings to contribute to responsible functioning of these trials and adequate protection of participants.

## Background

Evaluation of interventions in conjunction with their rollout is possible with stepped-wedge cluster randomized trials (SW-CRTs), which randomly allocate clusters (e.g., communities, hospitals, or entire health systems) sequentially to the intervention until all clusters are exposed [1]. Stepped-wedge trials are forms of cluster randomized trials (CRTs) that raise important ethical issues which are different from those of conventional CRTs. There are two forms of SW-CRTs. They may take the form of a cohort in which the same participants within clusters are being followed over time and crossover between interventions is at both the subject and cohort levels. They may also be cross-sectional studies, in which new participants are included after each step and crossover of the intervention only takes place at the cluster level [2]. The difference between the two types is morally relevant because in cross-sectional SW-CRTs not all participants will receive the intervention.

Because SW-CRTs are cluster randomized trials, in principle the same ethical conditions apply as for cluster randomized trials, such as those laid down in the Ottawa Statement on the Ethical Design and Conduct of Cluster Randomized Trials (2012) and the Council for International Organizations of Medical Sciences (CIOMS) International Ethical Guidelines for Health-related Research Involving Humans (2016) [3, 4]. However, because morally relevant differences exist between CRTs and SW-CRTs, ethical principles may require special attention when applied to the context of SW-CRTs.

First, classification of use of the design is morally important. SW-CRT designs are used in particular to evaluate the implementation of interventions. Use of these designs to evaluate implementation of interventions can lead to questions about whether the evaluation constitutes research or local service evaluation. SW-CRTs that are not classified as research do not fall under ethical guidance on human participant research and may not require research ethics committee (REC) review, informed consent, or trial registration. Others have identified that in SW-CRTs more generally, the studies are often not recorded with a trial registration database, and a minority also do not undergo ethical review [5].

Second, researchers and members of RECs should pay special attention to the justification of the design, in particular to clinical equipoise, meaning whether there is sufficient disagreement among health care professionals about the relative merits of the alternatives under study [2]. One of the reasons often put forward as justification for using the SW-CRT design is that the intervention “has [been] shown to be effective in more controlled settings” [6] or “there is a prior belief that the intervention will do more good than harm, rather than a prior belief of equipoise” [7]. Therefore, careful ethical discussion will be essential prior to the start of an SW-CRT to decide whether further evaluation of the effectiveness or safety of an intervention is warranted [2]. Furthermore, if it is felt that participants in the control arm should not be withheld from the intervention under study, it is important to acknowledge up front that even though all clusters will eventually receive the intervention, some clusters will receive it later than others, and, as described above, in cross-sectional types of SW-CRTs, not all individuals will receive the intervention. Consequently, in addition to ensuring clinical equipoise at the start of the trial, due consideration must be given to any consequences associated with this delay in the rollout to some clusters [8].

SW-CRTs are increasingly popular in health-related research [1] in both high- and low-resource settings [5]. Although the same ethical principles apply to the use of these designs in both settings [4], there may be specific ethical issues that researchers face when designing and conducting SW-CRTs in low-resource settings. Knowledge of these issues can help to improve the ethical conduct of these trials in a global health context. There is a theoretical reason to assume that the context of low-resource settings, characterized by factors identified by Ezekiel Emanuel and colleagues such as “poverty, limited health-care services, illiteracy, cultural and linguistic differences, limited understanding of the nature of scientific research, … [less well-established] regulatory infrastructures and independent oversight processes” [9], may influence how the two ethical issues that we identified are interpreted. At the same time, whether SW-RCTs do raise specific ethical issues that are not seen in the context of high-income country research will be part of our investigation.

## Summary relevant ethical guidance

The Ottawa Statement (2012) and the CIOMS guidelines (2016) provide relevant guidance for the design and conduct of CRTs. The Ottawa Statement has identified seven ethical issues that should be taken into account when ethically evaluating trials using a cluster design:

1. Justifying the cluster randomized design
2. Ensuring appropriate REC review
3. Identifying research participants
4. Obtaining informed consent
5. Role and authority of gatekeepers
6. Assessing benefits and harms
7. Protecting vulnerable participants

These issues are also mentioned in the CIOMS guidelines, in particular in guideline 21. Although research in low-resource settings is a central issue in the CIOMS guidelines, and although both guidelines address ethical issues for CRTs, neither document provides guidance specific to SW-CRTs, let alone SW-CRTs conducted in low-resource settings.

## Ethical issues posed when conducting SW-CRTs in low-resource settings

In line with the ethical issues set out in the Background section above—classification and justification of the design—here we use two case studies to illustrate these issues in the context of low-resource settings. In November 2017, the Global Forum on Bioethics in Research (GFBR) held their annual meeting in Bangkok, Thailand. The 2017 meeting focused on the ethics of alternative trial designs, including SW-CRTs conducted in low-resource settings. The cases presented by participants at the GFBR meeting were taken as a starting point for this paper. The cases were carefully selected by a GFBR planning committee and received mentoring support of planning committee members before presentation at the meeting. This paper focuses on the ethics of SW-CRTs by analyzing and discussing the two case studies presented at the GFBR meeting.

We first consider whether the SW-CRTs in our case studies should be classified as research for the purposes of design, ethical approval, and trial registration. The second question for our case studies is the justification for the design and whether there is equipoise to perform a randomized trial. We also consider when and under what circumstances it is justifiable to delay rollout of the intervention to both clusters and participants. Finally, we assess whether and when it is justified for researchers to choose clusters to initiate the intervention at the first step, because of the need or desire to help more “needy” clusters.

## Case study 1: Que Vivan Las Madres

### Background

The neonatal mortality rate is high in Guatemala at approximately 22 per 1000 pregnancies [10], and in some districts, the rate is double the national figure. Mothers are often reluctant to give birth in health centers because there is a strong sense that the traditional birth attendant is integral to maternal care. In rural areas, approximately 75% of mothers give birth at home, and this is believed to contribute to the high neonatal and maternal mortality rates. Furthermore, when mothers do give birth in a health center, they often arrive late in the birth process, and the health centers are poorly equipped to deal with complex births. It has been hypothesized that encouraging more women to give birth in a health center, particularly when there are early signals that the birth is not going well, might help reduce the high neonatal and maternal morbidity and mortality. It is also hypothesized that enhanced skills training in the health centers might make health care providers better equipped to deal with difficult births.

### Study design

The Que Vivan las Madres (QVLM) study, translated to English as “long live mothers,” is a SW-CRT conducted from January 2014 to January 2017 by the Epidemiological Research Centre in Sexual and Reproductive Health in Guatemala. The aim of the study was to determine if a package of interventions could increase the number of women giving birth in a health center to help improve the delivery and care of complicated deliveries, thereby decreasing the rate of both neonatal and maternal morbidity and mortality. During the study, 33 health centers in 2 districts of Guatemala were divided into 6 groups based on geographical proximity and sequentially randomized to initiate a package of 3 interventions described below.

### Methodology

The unit of randomization in QVLM was groups of five or six adjacent municipalities. Health centers within these geographical areas were then allocated to 1 of 12 sequences that dictated the time at which the intervention would be rolled out to all health centers in their geographical area. This cluster evaluation was necessary because it made study implementation logistically easier and prevented contamination from some components of the intervention that were delivered across wide areas (i.e., media campaigns). The evaluation used randomization in part but was not a fully randomized design, because the first cluster to transition to the intervention was determined by logistic factors related to implementing the training packages. In addition, the investigators alternated allocation between the two regions to promote a sense of fairness in the order of the rollout.

The intervention package consisted of (1) a simulation-based training program for obstetric emergencies (Programa de Rescate Obstétrico y Neonatal: Tratamiento Óptimo y Oportuno [PRONTO]) [11]; (2) a social marketing campaign that included radio spots, posters in public facilities such as schools and health centers, and the distribution of other promotional materials; and (3) activities with traditional birth attendants aimed to improve links with institutional caregivers.

Extensive data collection procedures were established throughout the districts participating in the study. Patient data collection did not include personal identification information and focused only on the morbidity and mortality outcomes of birth care.

### Consent and ethical approval

Mothers were not asked for their consent to take part in the study; this meant that their consent was not obtained for the use of their data. Health care professionals consented to take part in the education activities, and they were unaware that they were participating in a study during the control period. During the intervention, the study was publicized by the social marketing campaign, and thus caregivers and patients were aware that the QVLM project was ongoing. Nonetheless, no details about the study design and methodology were made public by the social marketing campaign or the PRONTO training. Public health authorities approved the implementation of the evaluation. Ethical approval was obtained in 2014 from the San Francisco General Hospital Panel and National Ethics Committee of Guatemala. The study was retrospectively registered as a clinical trial (ClinicalTrials.gov, NCT03151070).

### Delayed rollout of the intervention that had promising effect

The PRONTO intervention is a culturally adapted training curriculum targeted at health care providers and traditional birth attendants. It has been shown to have potential beneficial effects in nonrandomized evaluations in other settings [12]. Other components of the intervention (media campaigns and integration of traditional birth attendants) had not been evaluated in this context. Thus, this package of interventions was developed and tailored to focus on local context-specific issues, but its fundamental components had demonstrated promising results in other settings [12, 13]. Although all districts eventually received the package of interventions, mothers who gave birth before their district had crossed over to the intervention condition did not.

## Case study 2: Atmiyata

### Background

Mental illness is a substantial public health burden in India. Approximately 70 million people in India experience some form of mental illness [14, 15]. Approximately 20% of the Indian population is affected by common mental health disorders (CMDs), such as anxiety and depression. People with mental health problems often face discrimination in their communities [16, 17], which can reduce willingness to seek help from mental health care providers. Supply side factors, such as the paucity of trained mental health professionals in India, means that there are insufficient human resources to address the burden of CMD in the community [18], particularly in rural areas [19].

At the village level, though community health workers and nonspecialists are available and provide health services, few are trained to detect or identify mental health problems. There are existing approaches to addressing mental health issues in India, largely through intervening in formal health care services in the public sector (i.e., training community health workers).

### Study design

The study is based in rural regions of the Mehsana district in the State of Gujarat, India. Mehsana has a rural population of approximately 1.5 million. “Atmiyata” (meaning “empathy” or “shared compassion”) is a community-based mental health intervention that seeks to support and build community-based volunteers to reduce mental health distress and increase access to community health resources. The intervention develops the capacity of volunteers to identify and provide basic, low-intensity counseling to people with CMDs [20]. An SW-CRT was conducted to determine how effective the Atmiyata intervention is in reducing symptoms associated with CMD when implemented on a large scale.

In this design, villages were grouped by geographical areas to form four clusters in the district of Mehsana, Gujarat, India. Each geographical area contained 14 primary health centers (PHCs), each providing care for approximately 12 or 13 villages. All clusters were initially observed under the control condition, and one cluster was randomly selected to transition to the intervention condition every fifth month. The trial started in April 2017 and ended in December 2018.

### Methodology

In the Atmiyata case study, the unit of randomization was the PHC. The PHC had been selected as the unit of randomization because the administrative organization of primary health care is such that there is almost no inter-PHC movement of people for health care, and thus contamination of the intervention can be avoided. The PHCs were randomly assigned to four sequences that dictated when each group of villages would receive the intervention. In this study, the first cluster of villages to receive the intervention was not decided at random. Rather, this decision was reached during the study launch meeting with district health officials, local politicians, and community leaders, at which it was suggested that the intervention should be rolled out to one specific cluster of villages before others. Thus, in the Atmiyata study, the first cluster to receive the intervention was chosen by policy makers and community leaders.

### Consent and ethical approval

Permission was obtained from the Department of Health for the State of Gujarat for project implementation and data collection. The health care practitioners were informed about the implementation of the intervention but were not informed about the SW-CRT itself. Before approaching the individual participants, the data collector informed the village head about the study and the purpose of the data collection. Written informed consent was obtained from each participant enrolled in the trial during the control and intervention phases. The information provided to each of these participants included the purpose of the study, benefits and risks for the participant, and information about withdrawal from the study. The Indian Law Society Ethics Committee approved the study (ILS/14/2017), and an additional ethical approval was obtained from the local ethics committee of the Hospital for Mental Health in Ahmedabad. The trial is registered prospectively with the Clinical Trials Registry – India under registry number CTRI/2017/03/008139.

### Delayed rollout of the intervention that had promising effect

The core of the Atmiyata program is delivering low-intensity counseling to people with CMDs via trained community volunteers. The counseling techniques used are those of active listening, behavioral activation, and problem solving. Several programs have been developed in recent years to build the capacity of community health workers or primary care health workers or both, with the aim of increasing their uptake of mental health tasks [21–23]. Research has demonstrated the potential efficacy of such initiatives in India as well as in other parts of South Asia [24–26]. A Cochrane review on nonspecialist lay mental health worker interventions concluded that although evidence exists on the impact of lay workers on mental health, more research is needed on the type of lay worker, the intervention, and their effectiveness [27]. There is also evidence that lay health care workers can deliver problem-solving therapy to people with CMDs [28]. The Atmiyata intervention underwent a pilot evaluation [20]. Thus, there was considerable evidence, albeit not context-specific and high-level, of the effectiveness of the Atmiyata intervention.

## Ethical evaluation

### Classification of the design: is it research?

If an SW-CRT is not considered as research and rather is subsumed under the umbrella of quality improvement activities of health care processes within a particular organization, then it might be appropriate not to obtain ethical approval and not to register the study with a trial registration database. However, the underlying objective to provide a robust evaluation of the effectiveness for generalized dissemination makes the SW-CRT no less a research study than any other form of randomized trial. In general, SW-CRTs should all be registered in a trial database, and all should undergo ethical review and oversight, not only to protect the liberty and welfare interests of participants but also to guard against publication bias.

The QVLM study and the Atmiyata study should be classified as research and not as service evaluations, because both are designed to provide generalizable evidence for wider contexts. Both studies underwent ethical review. However, the QVLM study should have been registered with a trial database.

### Delayed rollout of an intervention: is it justifiable to delay the rollout of an intervention that has promising effect?

SW-CRTs are often portrayed as a method of evaluation of an intervention that is “likely to work” or has “perceived effectiveness.” However, in situations in which there is no clear robust evidence that the intervention is effective in the local setting, and when the intervention has the potential to be harmful (even if only by wasting resources), then we argue that under the paradigm of evidence-based medicine, there is a reasonable ground to conduct a randomized evaluation. In some situations, there may be good evidence that the intervention works in other settings, and this evidence might even be perceived to be generalizable, but limited resources in low-resource settings might mean that the intervention is not available. In such circumstances, SW-CRTs may be used to satisfy local policy makers by providing local evidence of effectiveness while respecting those who participate in the randomized evaluation by making the intervention available to all (either all clusters or all participants) at some point. Adding local evidence of effectiveness might be seen as an insufficient basis for initiating SW-CRTs; others may argue that the gap in service delivery and health budgets in low-resource settings is huge. They argue that low-resource settings need low-cost, effective, scalable, and sustainable interventions. However, SW-CRTs can provide an opportunity to provide a rigorous evaluation of interventions destined to be rolled out on the basis of limited evidence [8]. Further ethical evaluation will be essential to provide a full solution to this dilemma.

Delayed rollout of the intervention in both studies analyzed turns out not to have been morally problematic, because evidence of effectiveness of the interventions under evaluation was limited for both.

### How pragmatic is pragmatic?

The SW-CRT design is viewed as pragmatic because it can provide a means of conducting a randomized evaluation within a naturalistic setting, the only requirement being that the order of the rollout is randomized. Yet, how pragmatic should SW-CRTs be? Should they be so pragmatic as to allow the order of the rollout to be dictated not at random but using an order imposed by other stakeholders? To provide randomized evidence and to generate evidence on par with other randomized trials, SW-CRTs should use random allocation and not follow the order dictated by stakeholders [29]. When it is not possible to randomize the order of the rollout due to any constraints imposed in the context of the evaluation, a randomized evaluation is not possible, and researchers should look for other methods of evaluation. If there is sufficient evidence of the effect of the intervention (e.g., limited generalizability of health systems interventions from one health system to another), however, direct implementation without further evaluation should be considered [2].

Both case studies were only partially randomized insofar as both argued that “needier” clusters should receive the intervention first and thus were not part of the randomized rollout. This is problematic because it jeopardizes the causal inference that can be drawn from randomized evaluations.

## Discussion

The design and conduct of SW-CRTs raises at least two ethical issues that need special consideration in both high- and low-resource settings: the classification of this design as research or nonresearch and the justification for the design. This justification should include reasons supporting the (randomized) delay in the rollout of the intervention for some clusters and participants, exposing all clusters to the intervention, and the staggered rollout of the intervention.

In both case studies situated in low-resource settings, we did not find different interpretations of ethical principles and issues that are also encountered in high-income settings. A specific issue we found in both case studies was that pressure among stakeholders exerted influence on the order of the rollout, so neither study had a truly randomized design. Those clusters selected to receive the intervention first might have been systematically different from those clusters not selected. Randomization grounds causal inferences. So, in SW-CRTs in which the order of the rollout is not determined at random, causal inferences cannot be made, unless those clusters are excluded from the trial analysis. Methodological flaws such as this one therefore reduce the social value of studies. Given the pressures to adopt interventions in settings with high need, it is important for researchers, funders, and politicians not to act to diminish the quality of the science in these studies.

A second specific ethical issue we encountered, and one already reported by others [5], was that researchers should acknowledge the importance of registration of the SW-CRT. However, we cannot deduce from these specific ethical issues that they are unique to the conduct of SW-CRTs in low-resource settings. They may also occur in high-income settings.

### Broader context

In common with other CRTs, there are ethical issues that SW-CRTs raise, which we have not considered here [30, 31]. In common with conventional CRTs, these issues include gatekeeping, identifying the research participant, and obtaining informed consent.

Perhaps the most pertinent of these is, Who are the research participants in this study? SW-CRTs often evaluate cluster-level interventions, and just who are the research participants may not be obvious. The Ottawa Statement elucidated some of these issues for parallel cluster trials. However, the identification of the research participant in cluster trials is known not to be straightforward. Health care providers are rarely identified as the research participants. As is common with the case studies considered here, the research participants in evaluations of interventions that involve training of health care providers will often be the health care providers themselves. There are, of course, many questions that follow from this, including whether the health care providers should be free to refuse the training and whether their informed consent is required.

There are also other broader issues of consent. For example, the Ottawa Statement recommends that researchers “get consent where possible” in cluster trials, and this commonly means obtaining consent for some study procedures (such as data collection) but not others (such as exposure to the intervention) [3]. Further, it argues that a waiver of consent is commonly appropriate for cluster-level interventions. In most studies, it might be feasible to seek consent for differing components of the trial (e.g., use of their data) [30]. If both health providers and women in labor are research participants, to what should each group have consented? Furthermore, issues still need to be resolved regarding identification of the gatekeeper when clusters consist of groups of villages and an intervention is delivered at the village level but randomization happens at the cluster level [3]. Similarly, there is no clear procedure for the type of consent that is required in these circumstances.

## Conclusion

The case studies we analyzed were classified as research, and the use of an SW-CRT design did not seem to raise special ethical scrutiny apart from some minor issues for improvement. Although the focus of our case study analysis has been on the conduct of these trials in low-resource settings, our analysis has not demonstrated that ethical issues of SW-CRTs are different in low- or high-income settings. At the same time, in the cases we analyzed, the questions of whether and how to conduct the proposed SW-CRT also turned out to be a political issue. Further ethical evaluation will be essential to answer whether the conduct of SW-CRTs should be considered as an appropriate means to meet the health interests and needs of communities in low-resource settings. We hope that this evaluation will result in specific ethical guidelines for the use of SW-CRTs in both high- and low-resource settings to contribute to responsible trial conduct and adequate protection of trial participants.

## Data Availability

Not applicable.

## Abbreviations

CIOMS: Council for International Organizations of Medical Sciences
CMD: Common mental health disorder
CRT: Cluster randomized trial
GFBR: Global Forum on Bioethics in Research
PHC: Primary health center
PRONTO: Programa de Rescate Obstétrico y Neonatal: Tratamiento Óptimo y Oportuno
QVLM: Que Vivan las Madres
REC: Research ethics committee
SW-CRT: Stepped-wedge cluster randomized trial
